# Preparation and Properties of a Novel Microcrystalline Cellulose-Filled Composites Based on Polyamide 6/High-Density Polyethylene

**DOI:** 10.3390/ma10070808

**Published:** 2017-07-16

**Authors:** Shihua Xu, Shunmin Yi, Jun He, Haigang Wang, Yiqun Fang, Qingwen Wang

**Affiliations:** 1 Key Laboratory of Bio-based Material Science and Technology (Ministry of Education), Northeast Forestry University, Harbin 150040, China; xush@nefu.edu.cn (S.X.); binshanmu@nefu.edu.cn (S.Y.); hgwang@nefu.edu.cn (H.W.); 2 Nanjing Xuhua Sundi New Building Materials Co., Ltd., Nanjing 211224, China; xush@nefu.edu.cn; 3 College of Materials and Energy, South China Agricultural University, Guangzhou 510642, China

**Keywords:** lithium chloride, microcrystalline cellulose, polyamide 6/high-density polyethylene, melting point

## Abstract

In the present study, lithium chloride (LiCl) was utilized as a modifier to reduce the melting point of polyamide 6 (PA6), and then 15 wt % microcrystalline cellulose (MCC) was compounded with low melting point PA6/high-density polyethylene (HDPE) by hot pressing. Crystallization analysis revealed that as little as 3 wt % LiCl transformed the crystallographic forms of PA6 from semi-crystalline to an amorphous state (melting point: 220 °C to none), which sharply reduced the processing temperature of the composites. LiCl improved the mechanical properties of the composites, as evidenced by the fact that the impact strength of the composites was increased by 90%. HDPE increased the impact strength of PA6/MCC composites. In addition, morphological analysis revealed that incorporation of LiCl and maleic anhydride grafted high-density polyethylene (MAPE) improved the interfacial adhesion. LiCl increased the glass transition temperature of the composites (the maximum is 72.6 °C).

## 1. Introduction

Over the last two decades, studies about polymer blends based on polyamide 6 (PA6) and high-density polyethylene (HDPE) have received a great deal of interests. As an engineering plastic, PA6 is widely used in engineering domains for its excellent rigidity, thermal stability, and high mechanical performance. However, high cost and brittleness have limited its application. On the other hand, as one of the most promising common plastics, HDPE has many advantages, including low cost, high impact strength, good processability, and significant damping. However, HDPE has some inherent weaknesses, such as low heat deflection temperature and weak rigidity. Thereby, blends of PA6 and HDPE may result in polymers with a synergic combination of these properties to overcome their disadvantages [1,2,3,4,5,6,7,8].

Although polymers based on petroleum are in widespread use all over the world, what follow are sharp environmental issues. Bio-based materials are regarded as having some of the greatest potential to replace and reduce the depletion of fossil resources [9,10], which is due to the unique properties of natural fibers, including biodegradability, renewability, high mechanical properties, low cost, and density [11]. In contrast with other natural fibers, microcrystalline cellulose (MCC) with high specific surface area and crystallinity has received much attention, where the amorphous regions have been removed by hydrolysis process [11,12]. 

The thermodynamical immiscibility of PA6 and HDPE leads to poor interfacial adhesion. Thereby, a large number of studies focused on interface issues by using various compatibilizers, such as maleic anhydride grafted with high-density polyethylene (MAPE), styrene-b-ethylene-co-butylene-b-styrene grafted with maleic anhydride (SEBS-g-MA), and ethylene-b-glycidyl methacrylate (E-GMA), etc. [13,14,15]. However, it is very difficult to prepare natural fibers reinforced with PA6/HDPE blends, due to the conflicts of the high melting point of PA6 and the low degradation temperature of natural fibers. Some efforts were considered to figure out this problem. Reducing the melting point of polyamides is an effective method, and an increase in the number of papers indicates that small amounts of lithium chloride (LiCl) could decrease the melting point of PA6; then PA6 and wheat straws were blended together to prepare PA6/wheat straw composites. The results indicated that LiCl increased the tensile and flexural modulus, but decreased the impact strength, the tensile strength, and the flexural strength [16,17,18]. 

In this paper, LiCl was used to decrease the melting point of PA6 and the processing temperature, and improve the mechanical properties of the composites. HDPE was used to improve the impact strength of the composites. The effects of various LiCl and MAPE contents on crystal, mechanical, morphological, and thermal properties of the resulting composites were studied.

## 2. Materials and Methods

### 2.1. Materials

Commercially-available PA6 (1013b) with a density of 1.14 g/cm^3^ and a melt flow index of 22 g/10 min (235 °C, 2.16 kg), was purchased from UBE Industries (Tokyo, Japan). LiCl (analytical reagent), supplied by Rgent Industries Ltd (Tianjin, China)., was applied to decrease the melting point of PA6. HDPE (5000S) was purchased from Daqing Petrochemical (Daqing, China) with a density of 0.954 g/cm^3^ and a melt flow index of 0.7 g/10 min (190 °C, 2.16 kg according to ASTM D1238). MAPE (A-C^®^ 575A) was supplied by Honeywell International (Shanghai, China) with a maleic anhydride grafting ratio of 3 wt %. MCC (food grade) was purchased from Hongyuan Food Additives (Guangzhou, China) with the effective substance of 99%. The MCC was a powder with a particle size range from 26 µm to 96 µm and the average particle was 50 µm.

### 2.2. Sample Preparation

Prior to compounding, PA6, MCC, and LiCl were dried at 103 °C for 12 h in an oven, and LiCl was ground into particles that passed through a 100-mesh screen; then PA6, HDPE, MAPE, and LiCl were blended in a Haake torque rheometer (Thermo Fisher Scientific, Waltham, MA, USA) at 230 °C for 5 min with the speed of 50 rpm (The blends of PA6, HDPE, MAPE, and LiCl was abbreviated as the plastics blends in the context). The variation of two components was considered: (1) the weight ratio of PA6, HDPE, and MAPE was kept at 60/30/10. The LiCl content in the plastics blends was 0%, 0.5%, 1.0%, 1.5%, 2.0%, 2.5%, and 3.0%; and (2) The LiCl content in the plastics blends was kept at 1.0%. The weight ratio of PA6, HDPE and MAPE was 60/35/5, 60/25/15, and 60/20/20. The plastic blends were crushed into granules in the crushing mill, respectively. 

The plastics blends and MCC with the weight ratio of 85/15 were mixed in a Haake torque rheometer (Thermo Fisher Scientific, Waltham, MA, USA) at 230 °C for 5 min with the speed of 50 rpm. Subsequently, the blends were hot-pressed at 10 MPa for 10 min. To investigate the effect of HDPE on the composites, PA6/MCC composites was prepared. PA6 and MCC with the weight ratio of 85/15 were mixed in a Haake torque rheometer at 230 °C for 5 min with the speed of 50 rpm. Then the blends were hot-pressed at 10 MPa for 10 min. To reduce the energy consumption, the processing temperature was set to 20 °C above the melting point which tested by differential scanning calorimetry (DSC). For the composites with no melting point, the processing temperature was set to 200 °C (Table 1).

### 2.3. Characterization

#### 2.3.1. Differential Scanning Calorimetry

The non-isothermal crystallization and melting process of the composites were analyzed using Differential Scanning Calorimetry (DSC) (Pyris6, Perkin Elmer, Waltham, MA, USA) with a sample weight of 4–6 mg. All samples were heated to 230 °C and held for 10 min to wipe out the thermal history, and then cooled to 60 °C with the cooling rate of 10 °C/min, finally heated to 230 °C at the same rate under a nitrogen atmosphere. 

#### 2.3.2. Thermogravimetric Analysis

Thermogravimetric analyses were carried out with PE Instruments equipment (STA 6000-SQ8, Waltham, MA, USA ). Samples with the weight of 10 ± 0.5 mg were heated from 50 to 600 °C at a constant rate of 10 °C/min under a nitrogen atmosphere. 

#### 2.3.3. Mechanical Testing

Flexural and tensile tests were performed using an electromechanical universal testing machine (CMT5504, MTS, Shenzhen, China). According to ASTM D790 (Standard Test Methods for Flexural Properties of Unreinforced and Reinforced Plastics and Electrical Insulating Materials), the samples with dimension of 50 × 12.7 × 1.5 mm were prepared for the flexural tests. The test was carried out at a speed of 2 mm/min with a support span of 25.4 mm. According to ASTM D 638 (Standard Test Method for Tensile Properties of Plastics), dumbbell-shaped samples with the dimension of 115 mm in length, 19 mm in overall width, 6 mm in narrow width, and 1.5 mm in thickness were used for tensile tests, and the crosshead speed was set to 5 mm/min. Charpy impact tests of unnotched specimens were carried out with an impact machine (XJ-50G, Chengde, China) according to the Chinese standard (GB/T 1043). The dimension was 80 × 10 × 3 mm. All the specimens were performed in the condition of 23 ± 2 °C and 50 ± 5% (Relative Humidity). At least five specimens were tested for each formulation.

#### 2.3.4. Morphological Analysis

Cryo-fractured surfaces were generated by breaking the composites under liquid nitrogen conditions and subsequently sputter-coated with gold. The fractured surfaces were observed with a scanning electron microscope (QUNGTA200, FEI, Oregon, USA) at an accelerating voltage of 10 kV.

#### 2.3.5. Dynamic Mechanical Analysis

Dynamic mechanical analyses (DMA) of all samples were tested in three-point bending mode with Dynamic Mechanical Analyzer (Q800, TA, New Castle, USA). Rectangular specimens with a dimension of 35 × 12 × 1.5 mm were used. Experiments were carried out in the temperature range from −20 to 120 °C with a heating rate of 5 °C/min and a constant frequency of 1 Hz.

## 3. Results and Discussion

### 3.1. Differential Scanning Calorimetry Analysis

**Figure 1:**
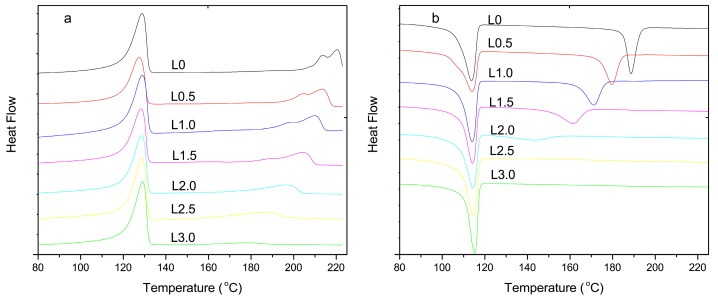
Differential Scanning Calorimetry (DSC) thermograms of composites with different LiCl contents: (**a**) melting curve; and (**b**) crystallization curve.

As the contents of LiCl increased, *T_p_*, *T_m_*, and *X_c_* of the composites declined (Figure 1 and Table 2). This was believed to be due to the break of hydrogen bonds between molecular chains by complexation of LiCl and amide groups [17]. The complexation restricted the motion of PA6 molecular chains, hence, the crystallization time showed the opposite trend (Table 2). *T_p_*, *T_m_*, and *X_c_* of the composites with 3 wt % LiCl were disappeared (Figure 1 and Table 2), which demonstrated that the crystal morphology of PA6 was varied from semi-crystalline to amorphous state.

### 3.2. Thermal Analysis

The peak temperature (*T_p_*) of the thermal degradation of MCC, PA6, and HDPE was given in Table 3. As can be seen, the peak temperature of MCC decreased in the LiCl content range of 0–2.0%, implying that LiCl could promote the degradation of MCC. Further increasing the LiCl content from 2.0% to 3.0%, *T_p_* of MCC did not have a substantial change. This result may indicate that there existed a critical concentration of LiCl. For PA6, the addition of LiCl significantly decreased the peak thermal degradation temperature, which is in agreement with the result that little LiCl could decrease the melting point of PA6. For HDPE, the incorporation of LiCl had a slight effect on the peak thermal degradation temperature.

### 3.3. Mechanical Analysis

**Figure 2:**
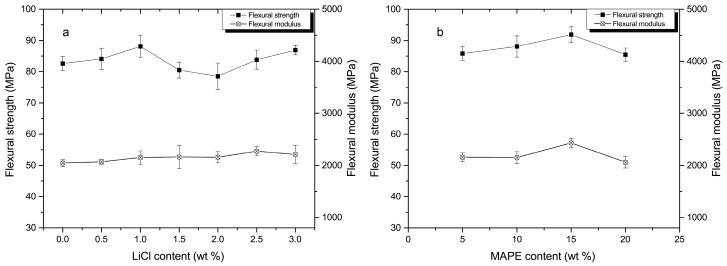
Flexural properties of composites: (a) in various LiCl contents; and (b) in various MAPE contents.

The flexural strength of composites was slightly improved with increasing of LiCl content to 1.0% (Figure 2a). The increase may be attributed to the formation of pseudo-crosslinking structure by the complexation of LiCl and amide groups [17]. Further increasing the LiCl content from 1.0% to 2.0%, the flexural strength of the composites decreased (Figure 2a). It may be due to the degradation of MCC promoted by LiCl (Table 3). However, the flexural strength of composites increased again when the LiCl content was above 2.0%. The reason may be that the effect of the complexation which offset the degradation of MCC. It can be proved by that the increase of LiCl content (above 2.0%) did not cause essential changes in the peak temperature of degradation (Table 3). LiCl prevented the motion of PA6 molecular chains and improved the stiffness of the composites, which can explain a slight increasing trend in the flexural modulus as the increase of LiCl content (Figure 2a). 

**Figure 3:**
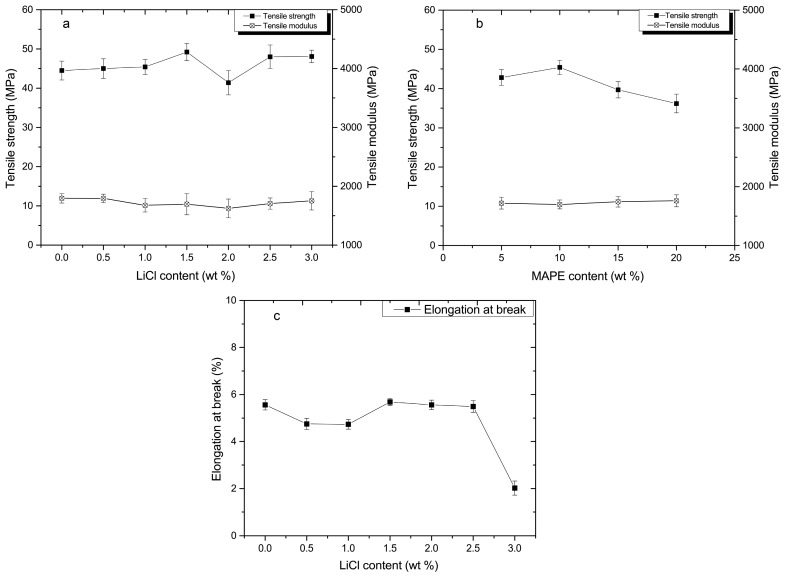
Tensile properties of composites: (**a**) tensile strength and modulus in various LiCl contents; (**b**) tensile strength and modulus in various MAPE contents; and (**c**) the elongation at break in various LiCl contents.

The composites displayed an increase in flexural strength and modulus in the MAPE content range of 5–15%, above which the flexural strength and modulus exhibited a decreasing trend (Figure 2b). As the compatibilizer, MAPE improved the interfacial adhesion and miscibility among PA6, HDPE, and MCC, where large amounts of the applied load may be transferred to the rigid PA6 and MCC. However, a multimolecular layer induced by excess MAPE (above 15%) was formed on the surface of MCC and PA6. It is difficult to bear further external loads due to the multimolecular layer of MAPE [19]. Furthermore, as the polar polymer, excess MAPE (above 15%) tended to aggregate and the formation of stress concentration decreased the flexural strength. The similar phenomenon that compatibilizers create a critical concentration have been observed in the literature [20]. The tensile strength of the composites exhibited the similar trend to the flexural strength, and the composites showed that the hardly-changed tensile modulus with the increase of LiCl and MAPE (Figure 3a,b). The composites revealed no obvious change in elongation at break with an increase of the LiCl content to 2.5%. With a further increase of the LiCl content (3.0%), the elongation at break of the composites was decreased significantly (Figure 3c). 

**Figure 4:**
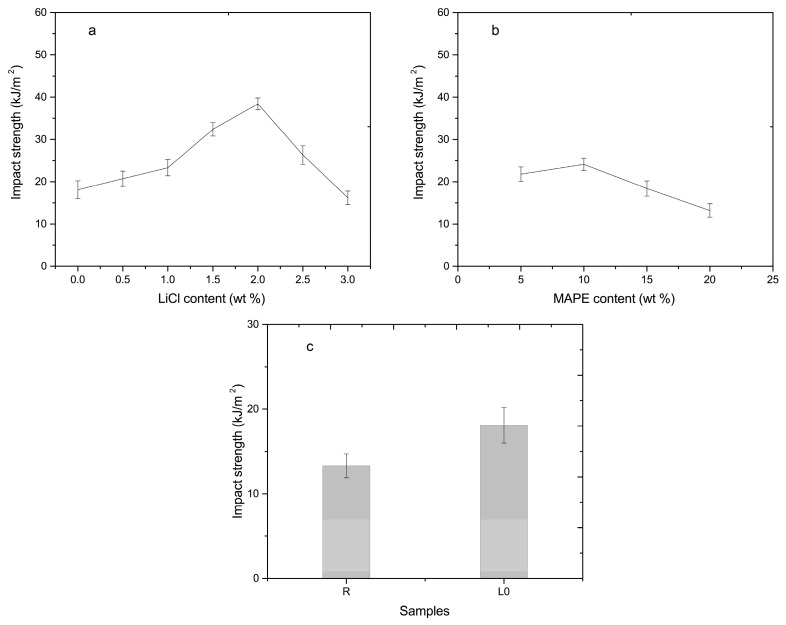
Impact properties of composites: (**a**) in various LiCl contents; (**b**) in various MAPE contents; and (**c**) PA6/MCC composites and PA6/HDPE/MCC composites without LiCl.

**Figure 5:**
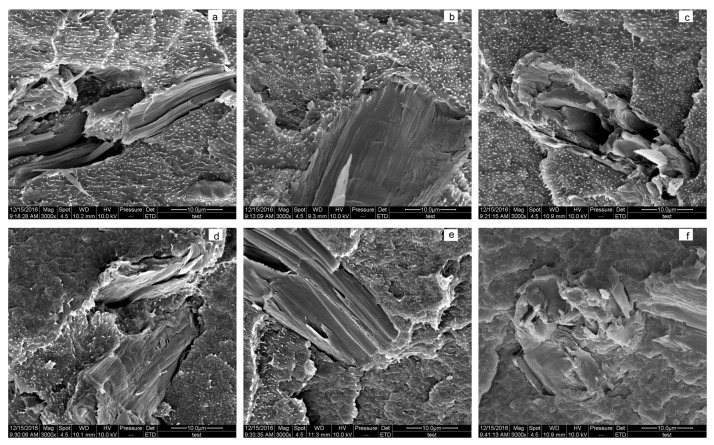

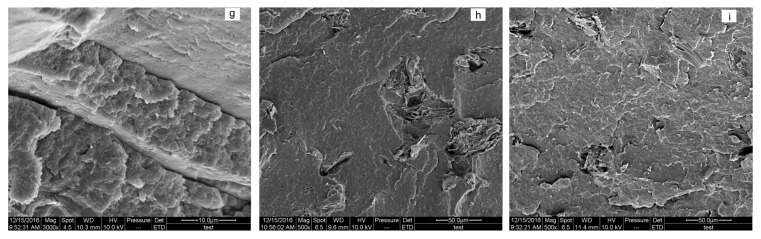
Morphologies of the composites in various LiCl contents and scale bar: (**a**) 0% (10 µm); (**b**) 0.5% (10 µm); (**c**) 1.0% (10 µm); (**d**) 1.5% (10 µm); (**e**) 2.0% (10 µm); (**f**) 2.5% (10 µm); (**g**) 3.0% (10 µm); (**h**) 0% (50 µm); and (**i**) 2.0% (50 µm).

**Figure 6:**
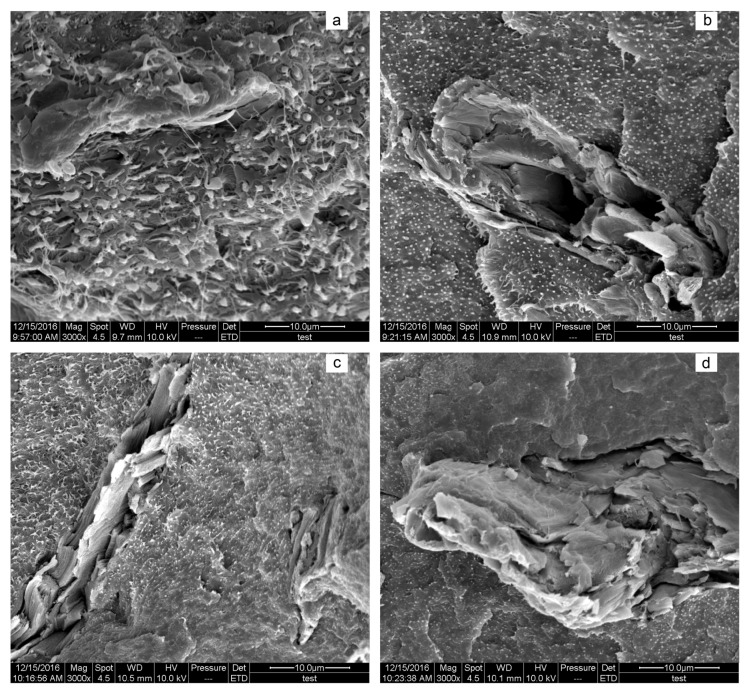
Morphologies of the composites in various MAPE contents: (**a**) 5%; (**b**) 10%; (**c**) 15%; and (**d**) 20%.

The impact strength of composites increased in the LiCl content range of 0–2.0%, and then decreased with a further increase in the LiCl content (Figure 4a). The composites gained an increase of 90% in the impact strength at a LiCl content of 2.0%. In principle, rigid ionic bonding between PA6 chains with the addition of LiCl went against the stress dissipation, the external energy would be consumed during crack propagation by unstable crack growth mechanisms. As a result, the impact strength was decreased [15]. However, the increase of interfacial compatibility promoted by LiCl between PA6 and HDPE made the impact energy transferred and dissipated from rigid PA6 to flexible HDPE (as will be shown later by SEM analysis), which prevailed over the positive effect of rigid ionic bonding in the LiCl content range of 0–2.0%. Increasing the LiCl content did not cause a substantial change of interfacial compatibility (above 2.0%) (Figure 5). Hence, the effect of rigid bonding on energy dissipation may be dominant. This result may explain the decreasing impact strength (above 2.0%). Compared to the composites with 5.0% MAPE, the impact strength of the composites with 10.0% MAPE was improved 10.6%, and then gradually decreased with the increasing of MAPE (Figure 4b). An increase of the impact strength was ascribed to better interfacial adhesion between PA6 and HDPE (Figure 6), where the impact energy was dissipated via HDPE [6]. The decrease may be attributed to the following two reasons: (1) the weight ratio of HDPE decreased as the content of MAPE was increased, and there were no sufficient flexible polymers to absorb and dissipate the external energy; (2) the abovementioned formation of the multimolecular layer and stress concentration contributed to the decrease.

The impact strength of PA6/HDPE/MCC composites increased by 36% compared to PA6/MCC composites (Figure 4c), which was ascribed to energy dissipation of flexible HDPE [6].

### 3.4. Morphology Analysis

**Figure 7:**
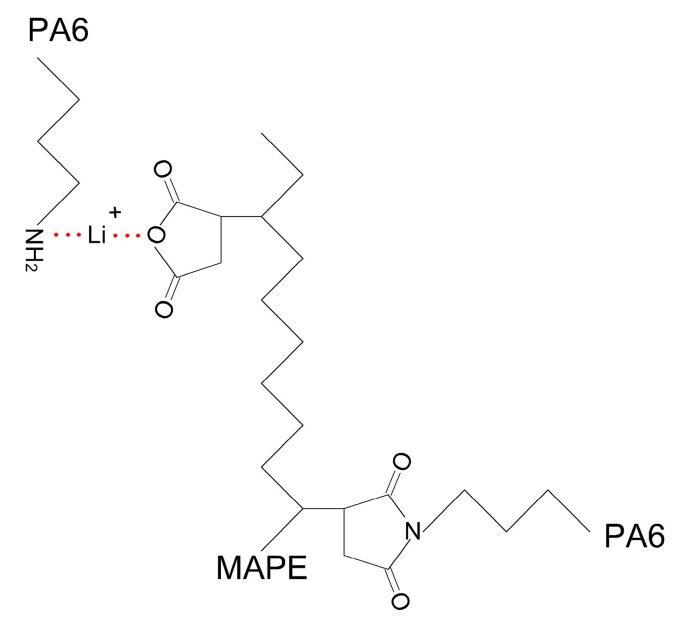
Scheme of complexation between LiCl and O or N.

In the composites without LiCl, large amounts of HDPE were dispersed in the continuous phase of PA6 in the form of stripe, strap, and other irregular shapes in the length range of 1 to 5 µm (Figure 5a), implying that although MAPE was applied as the compatibilizer, there was still weak interfacial adhesion between PA6 and HDPE with a MAPE content of 10%. The shapes of HDPE shifted toward much smaller globular particles in size in the PA6 matrix with a LiCl content range of 0.5–1.0% (Figure 5b,c). With the further increase of LiCl, the globular particles were diminished, and then vanished (Figure 5d–g). The above observation obviously disclosed that LiCl was in favor of interfacial adhesion between PA6 and HDPE, which may be explained by the following two reasons: (1) lithium cations of electron deficiency complexed with O or N of both amide groups and anhydride groups. As a result, more molecular chains of MAPE and PA6 were connected via LiCl (Figure 7); and (2) amorphous PA6 chains were increased with the increase of LiCl, it was easier for anhydride groups with high steric hindrance to penetrate into the inside of amorphous PA6. Consequently, the interfacial compatibility was improved by more chemical reaction between end amino groups of PA6 and anhydride groups of MAPE [21].

Figure 5h,i indicated that MCC was uniformly distributed in the composites, and MCC was broken rather than pulled out. A rougher surface and more cracks were observed in the composites with 2.0% LiCl than the composites without LiCl. This further proved that LiCl improved the impact strength via the energy dissipation mechanism [6].

In contrast with the composites at the MAPE content of 5%, the particle size of HDPE was reduced by one to two orders of magnitude for the composites with 10% MAPE, indicating that MAPE had the positive effect on compatibility between PA6 and HDPE. With the further increase in MAPE content, there was no significant change in the particle size of dispersive phase, except that the amounts of particles diminished slowly.

### 3.5. Dynamic Mechanical Analysis

**Figure 8:**
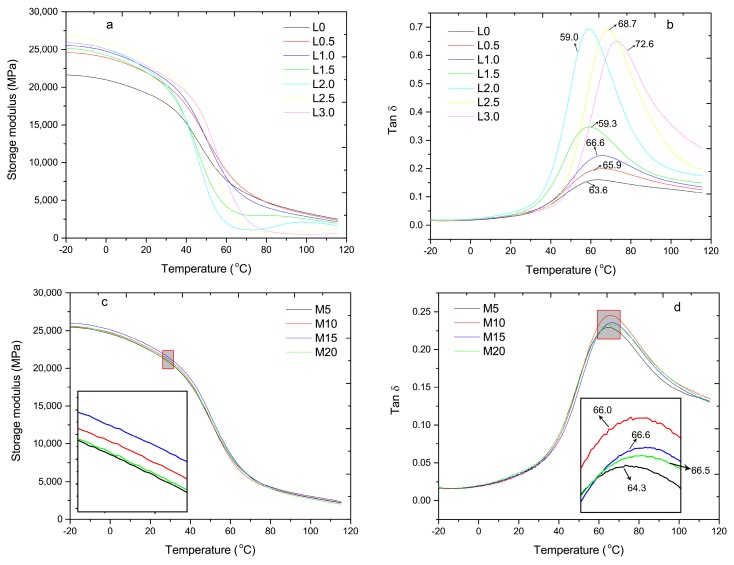
Dynamic mechanical properties for composites: (**a**) the storage modulus (E’) in various LiCl contents; (**b**) the loss factor (Tan δ) in various LiCl contents; (**c**) the storage modulus in various MAPE contents; and (**d**) the loss factor in various MAPE contents.

The storage modulus of the composites without LiCl was lower than those with LiCl below the room temperature (Figure 8a), which can be explained by the complexation of LiCl and amide groups that restricted the molecular chains’ motion of PA6. Above the glass transition temperature, the free volume of amorphous polymers will be expanded. Although it is impossible for the entire molecular chains to move, the thermal energy can overcome the barrier of internal rotation so that segmental movement of PA6 can be activated. As a result, the storage modulus was decreased. The more amorphous polymers, the lower the storage modulus, which can explain that the storage modulus of the composites was decreased with the increase of LiCl content above the glass transition temperature (Figure 8a). The storage modulus of the composites increased in the MAPE content range of 5–15%, and then decreased with a further increase in the MAPE content (Figure 8c), which was in agreement with flexural modulus (Figure 2b).

The incorporation of LiCl significantly affected the glass transition temperature of PA6. The glass transition temperature was improved by 3 °C in the LiCl content range of 0–1.0%, then was decreased in the LiCl content range of 1.5–2.0%. Further increase of the LiCl content (above 2.5%) caused an increase in the glass transition temperature (maximum was 72.6 °C) (Figure 8b). An increase could be attributed to the restriction of PA6 molecular chains motion via complexation of LiCl and amide groups. Furthermore, the hydrogen bonds between the hydroxy of MCC and amide groups of PA6 could be generated. Thereby, a sharp degradation of MCC had a negative effect on the glass transition temperature of PA6 (Table 3), which explained the decrease in the LiCl content range of 1.5–2.0%.

The composites demonstrated an increase of the peak width in tan δ with the increasing of LiCl (Figure 8b), which could be ascribed to the following reasons: (1) the miscibility and interfacial adhesion between PA6 and HDPE was improved via LiCl (Figure 5), which was usually observed in miscible polymer blends [22]; and (2) constraints of the molecular chains’ motion of PA6 resulted in an increase in the segmental relaxation time of PA6 [23]. The value of tan δ is related to the viscoelastic response of the materials. A higher tan δ of the composites suggested that the viscous nature was more dominant than the elastic property, as evidenced by the greater impact strength [24,25]. Tan δ of the composites was increased in the LiCl content range of 0–2.0%, and then was decreased with further increasing of the LiCl content (Figure 8b). This trend was consistent with the impact strength (Figure 4a). An increase in compatibility and interfacial adhesion between PA6 and HDPE confined the movement of PA6 chain, thereby, the glass transition temperature was increased (Figure 8d). In addition, tan δ of the composites and the impact strength followed the same trend with the increasing of MAPE. 

## 4. Conclusions

The results indicate that the addition of LiCl and HDPE is a novel and effective approach to increase the impact strength of PA6/MCC composites, as evidenced by the fact that the impact strength of composites was increased by 90% and 36%, respectively. Morphology analysis demonstrated both LiCl and MAPE have a positive effect on the interfacial adhesion of the composites. In addition, LiCl is in favor of the glass transition temperature of composites (the maximum is 72.6 °C).

## Figures and Tables

**Table 1:** Formulation and processing temperature of the composites.

Samples	LiCl (wt %)	MAPE (wt %)	Processing Temperature (°C)
L0	0	10	240
L0.5	0.5	10	235
L1.0	1	10	230
L1.5	1.5	10	225
L2.0	2	10	220
L2.5	2.5	10	210
L3.0	3	10	200
M5	1	5	230
M15	1	15	230
M20	1	20	230
R	0	0	240

LiCl (wt %): LiCl content in PA6/HDPE/MAPE/LiCl blends by weight; MAPE (wt %): MAPE content in PA6/HDPE/MAPE blends by weight (the weight ratio of PA6 was kept at 60%). Abbreviations of L0 - L3.0 represent the composites with the LiCl content of 0%, 0.5%, 1.0%, 1.5%, 2.0%, 2.5%, and 3.0%. Abbreviations of M5, M15, and M20 represent the composites with the MAPE content of 5%, 15%, and 20%. R represents PA6/MCC composites.

**Table 2:** DSC analysis data of composites with different LiCl contents.

Samples	*T_m_* (°C)	*T_p_* (°C)	Crystallization Time (s)	*X_c_* (%)
L0	220.4	188.6	47	20.0
L0.5	213.4	179.7	64	19.2
L1.0	209.9	171.1	78	17.8
L1.5	204.5	161.3	107	15.3
L2.0	197.0	143.3	169	10.9
L2.5	187.5	-	-	9.4
L3.0	-	-	-	-

*T_m_*: melting point of PA6; *T_p_*: peak temperature of crystallization of PA6; *X_c_*: crystallinity of PA6.

**Table 3:** Degradation temperature values derived from thermogravimetric analysis.

Samples	*T_p_* (°C)		
	Peak A	Peak B	Peak C
L0	366.2	442.5	482.7
L0.5	350.8	429.9	483.7
L1.0	343.0	422.9	480.8
L1.5	336.2	420.6	480.6
L2.0	319.8	418.5	479.8
L2.5	317.1	423.2	482.5
L3.0	314.6	421.0	483.1

*T_p_*: the peak temperature of degradation; the temperature of Peak A, peak B, and peak C are associated with the degradation of MCC, PA6, and HDPE, respectively.
